# Retroperitoneal Schwannoma: A Case Report and Diagnostic Approach to Retroperitoneal Masses

**DOI:** 10.7759/cureus.95658

**Published:** 2025-10-29

**Authors:** Nathalia Jácome-Pérez, Lina Charry Jiménez, Carlos Arias-Durán, Santiago Ramiréz-Figueroa

**Affiliations:** 1 Radiology, Universidad de Santander, Bucaramanga, COL; 2 Epidemiology and Public Health, Universidad Autónoma de Bucaramanga, Bucaramanga, COL; 3 General Medicine, Universidad Pedagógica y Tecnológica de Colombia, Bucaramanga, COL; 4 General Medicine, Universidad Industrial de Santander, Bucaramanga, COL

**Keywords:** benign abdominal mass, neurogenic tumor, retroperitoneal mass, retroperitoneal schwannoma, tumor imaging

## Abstract

Retroperitoneal masses represent a diagnostic challenge in imaging due to their uncommon location and variable presentation. Schwannomas are one of the possible etiologies. We report the case of a 50-year-old woman who presented to a tertiary referral hospital in Bucaramanga, Colombia, with chronic abdominal pain. Physical examination was unremarkable, but imaging revealed a giant retroperitoneal mass confirmed on contrast-enhanced abdominal computed tomography. The lesion showed neoplastic features, displacing the inferior vena cava and right kidney. The patient underwent surgical resection with radical lymphatic dissection, and histopathology confirmed a benign schwannoma. This case emphasizes the importance of considering schwannoma in the differential diagnosis of retroperitoneal tumors and illustrates the correlation between imaging and histopathologic findings.

## Introduction

Retroperitoneal tumors are rare; they account for less than 1% of all neoplasms and are often malignant, with sarcomas being the most common [1]. Among benign forms, retroperitoneal schwannomas represent only 0.5%-4% of cases [2], typically affecting women aged 20-50 years [3]. These slow-growing tumors often present incidentally or with non-specific symptoms and can mimic malignancy, requiring histopathological confirmation. They are usually encapsulated and show Antoni A areas (hypercellular, palisading nuclei, and Verocay bodies) and Antoni B areas (hypocellular with myxoid or cystic matrix) with strong S-100 and SOX10 expression [4]. Despite their benign nature, their rarity and non-specific features make them an important differential diagnosis for radiologists [5]. Imaging plays a crucial role in the detection, characterization, and preoperative assessment of these lesions, although findings can overlap with other retroperitoneal tumors, making radiologic evaluation challenging [6,7]. We report a giant retroperitoneal schwannoma successfully resected, with pathology confirmation and no evidence of recurrence on follow-up.

## Case presentation

**Figure 1:**
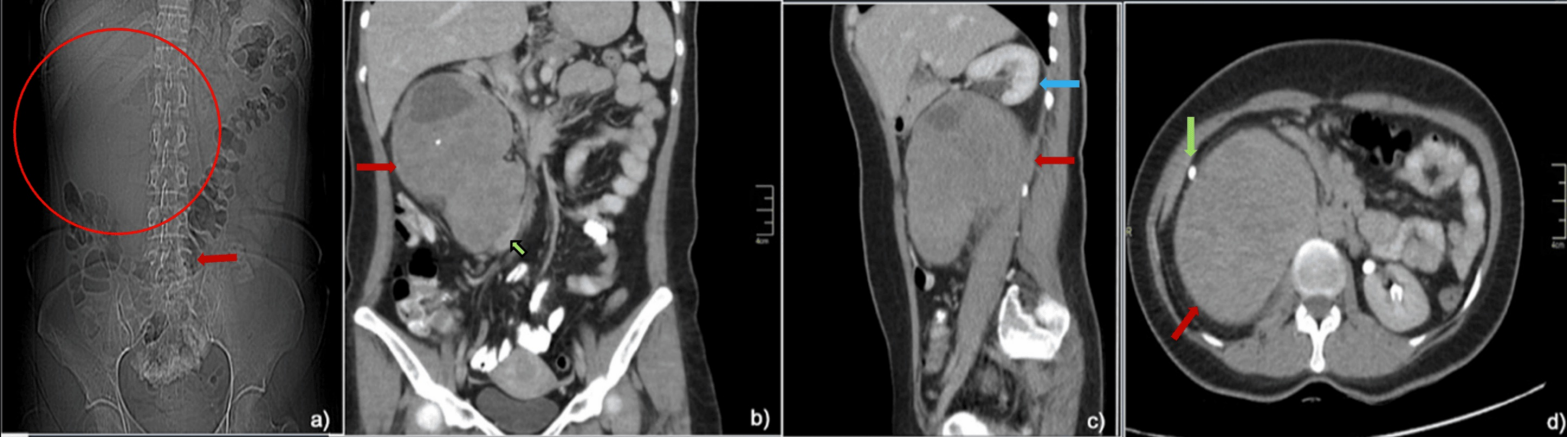
Contrast-enhanced abdominal CT (a) Localizer showing a space-occupying lesion in the right hypochondrium (outlined by a red circle) that displaces the hepatic flexure of the colon and the transverse colon toward the hypogastrium/mesogastrium (red arrow). (b) Right retroperitoneal mass (red arrow) with well-defined borders and heterogeneous density due to low-attenuation areas (which may correspond to areas with cystic degeneration) and a small calcification, causing medial displacement and compression of the inferior vena cava (green arrow). (c) Proximal and lateral displacement of the right kidney (blue arrow) caused by the retroperitoneal mass (red arrow). (d) Ipsilateral ureteral lateralization (green arrow). CT: computed tomography

**Figure 2:**
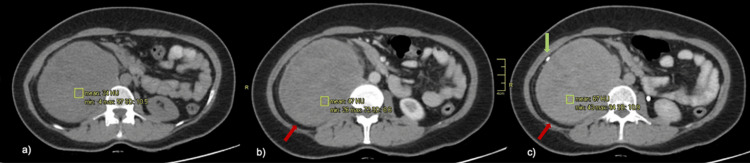
Contrast-enhanced abdominal CT characteristics of the mass The mass shows well-circumscribed borders with soft-tissue density and progressive post-contrast enhancement. Phases: (a) non-contrast phase (34 HU). (b) Arterial phase (47 HU), with the mass indicated by a red arrow. (c) Delayed phase (67 HU), showing the mass (red arrow) and right ureteral displacement caused by the lesion (green arrow). CT: computed tomography

**Figure 3:**
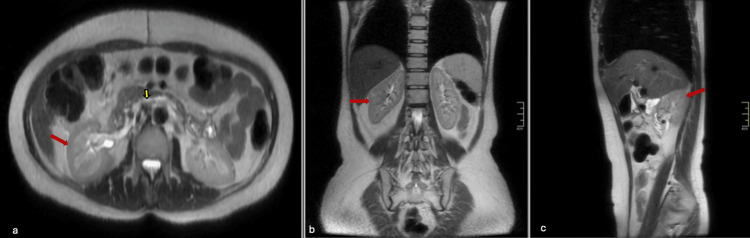
Follow-up non-contrast abdominal MRI Complete resection of the lesion. The right kidney (red arrow in (a-c)) has returned to its normal position within the renal fossa, showing normal size and enhancement pattern. The inferior vena cava (yellow arrow) is located in its normal retroperitoneal space adjacent to the aorta. No residual mass or abnormal soft tissue density is observed. MRI: magnetic resonance imaging

We present the case of a 50-year-old woman with chronic generalized abdominal pain of one year's duration and no significant medical history. Ultrasound revealed a hypoechoic retroperitoneal mass, further characterized on contrast-enhanced computed tomography (CT) as a right retroperitoneal lesion contacting and displacing cephalically the right kidney (Figures 1, 2). The initial differential diagnoses included a retroperitoneal leiomyoma, a nerve sheath tumor, or a sarcoma. She underwent surgery with radical lymph node dissection; histopathologic examination revealed a mesenchymal lesion with mild atypia, and immunohistochemistry demonstrated a low-grade spindle-cell tumor positive for S-100 with free margins, consistent with a benign schwannoma. Follow-up magnetic resonance imaging (MRI) at six months confirmed complete resection (Figure 3).

## Discussion

Retroperitoneal schwannoma is a rare neoplasm representing about 3% of all schwannomas and is most commonly found in the peripheral nerves of the neck, mediastinum, and extremities [8]. Its presentation is usually non-specific, including abdominal or lumbar pain, urinary or digestive disorders from mass effect, and palpable mass in large lesions; however, these tumors are often asymptomatic and may reach considerable size before producing symptoms, owing to the large and compliant nature of the retroperitoneal space [6]. In a recent clinicopathological analysis of 14 cases, the mean tumor diameter was 5.5 cm (range 2.5-9.5 cm), highlighting that many lesions remain moderate in size at diagnosis despite the potential for significant growth [7]. Importantly, tumor size alone is not a reliable predictor of malignancy in retroperitoneal schwannomas [9]. In this case, abdominal pain was the main clinical manifestation.

As for imaging findings, advanced cross-sectional imaging techniques, particularly CT and MRI, are required to achieve the most accurate characterization and optimal surgical planning of the mass. On ultrasound, they appear as solid, hypoechoic masses that are well defined and internally heterogeneous [10].

On contrast-enhanced CT, they are usually visualized as encapsulated lesions with a density slightly lower than soft tissue due to their myelin and fat content, with progressive enhancement ranging from homogeneous to heterogeneous, depending on the degree of cystic degeneration, bleeding, or calcification [11]. MRI is the most sensitive technique, showing iso- or hypointense lesions on T1 and hyperintense lesions on T2, with characteristic signs such as the target sign or fascicular sign. These characteristics, although suggestive, are not pathognomonic and always require histological confirmation [1,3,12,13]. In our case, the mass showed defined contours and heterogeneous enhancement with cystic areas and a small calcification on contrast-enhanced CT. These findings are consistent with previous reports describing well-circumscribed retroperitoneal schwannomas with progressive enhancement and variable cystic degeneration on CT and MRI [5,9,11,13]. Similar imaging appearances have been documented by Rajiah et al. and Goenka et al., reinforcing the value of imaging in identifying the neurogenic origin of these lesions [1,2].

The diagnostic approach to retroperitoneal masses should be systematic, starting with lesion composition (solid, cystic, fatty, or fibrous). Differential diagnoses include benign and malignant entities such as liposarcoma, leiomyosarcoma, neurogenic tumors, lymphoma, and metastases. Accurate differentiation requires integrating age, clinical presentation, imaging, and histopathology (Table 1).

**Table 1:** Differential diagnosis of retroperitoneal masses The table summarizes the typical age distribution, characteristic MRI findings, and key distinguishing features of the main differential diagnoses of retroperitoneal masses, including benign and malignant entities. MRI: magnetic resonance imaging; 18F-FDG PET/CT: fluorine-18 fluorodeoxyglucose positron emission tomography/computed tomography; DWI: diffusion-weighted imaging

Typical age	Differential diagnosis	MRI findings	Key points
20–50 y	Retroperitoneal schwannoma	Encapsulated mass, iso-/hypointense on T1, heterogeneous hyperintense on T2; target/fascicular signs	Presence of capsule
20–50 y	Neurofibroma	Hypointense on T1 and T2. May show myxoid degeneration. Contrast enhancement present	No capsule. Target sign. T2 hyperintense ring
10–29 y	Ganglioneuroma	Well-defined mass. T1 hypointense-T2 heterogeneous hyperintense	“Whorled sign” is considered typical of ganglioneuroma. This appears on T2-weighted imaging as a predominantly hyperintense tumor with focal low-signal and nodular aspects
40–70 y	Liposarcoma	Large heterogeneous mass with fat; T1 and T2 hyperintense, fat-suppressed on fat-sat. In dedifferentiated cases: calcifications and heterogeneous solid component	Most frequent retroperitoneal sarcoma. 18F-FDG PET/CT may assist in diagnosis
20–50 y	Paraganglioma	Hypervascular mass; heterogeneous T2, some with marked hyperintensity (“light-bulb sign”); intense enhancement. May show hemorrhage and necrosis	Consider in the context of catecholamine-related symptoms
40–60 y	Leiomyosarcoma	Large heterogeneous mass, possible central necrosis, potential vascular invasion	Highly aggressive, requires wide surgical resection
50–70 y	Retroperitoneal lymphoma	Multiple lymphadenopathies, isointense on T1, intermediate signal on T2, restricted diffusion (DWI)	Often multiple and non-encapsulated

In addition, some radiological signs have been described that help confirm its location and origin in a retroperitoneal organ, such as the “beak or claw” sign, the “phantom organ” sign, the “embedded organ” sign, and the “prominent feeding artery” sign [14]. Percutaneous biopsy is controversial due to risks such as hemorrhage, infection, rupture in cystic lesions, and potential tumor seeding [15]. Complete surgical resection remains the gold standard for retroperitoneal schwannoma, with 5- and 10-year survival rates of 75% and 45%, respectively. Retroperitoneal schwannomas are typically solitary, well-circumscribed, and non-invasive, allowing complete excision. However, resection can be technically complex due to the hypervascularity of the tumor and its proximity to neurovascular structures, which implies a risk of bleeding [16,17].

In our case, the retroperitoneal schwannoma was treated with complete surgical resection, and the diagnosis was confirmed by histopathology and immunohistochemistry. Follow-up MRI showed no recurrence, reinforcing the need for serial imaging, given recurrences, although rare, have been reported in up to 5%-10% of cases after incomplete resections [18]. The absence of recurrence during follow-up in our patient is consistent with previously reported cases, in which complete surgical excision was curative and recurrence was not observed [6,10].

## Conclusions

Retroperitoneal schwannomas are rare tumors that should always be considered in the differential diagnosis of retroperitoneal masses. Although histopathology remains the gold standard for definitive diagnosis, imaging plays a critical role in their initial characterization and clinical management. MRI findings such as well-defined margins, presence of a capsule, homogeneous enhancement, and signs like the target or fascicular sign are highly suggestive of schwannoma, even though degenerative changes including cystic areas, necrosis, or hemorrhage may also be present. Differentiation from other retroperitoneal tumors such as neurofibromas, ganglioneuromas, sarcomas, or paragangliomas relies on recognizing these radiologic patterns and correlating them with the clinical setting. Awareness of these features is essential for radiologists, as it can guide the diagnostic approach, reduce unnecessary interventions, and facilitate appropriate surgical planning.

## References

[1] Rajiah P, Sinha R, Cuevas C, Dubinsky TJ, Bush WH Jr, Kolokythas O (2011). Imaging of uncommon retroperitoneal masses. Radiographics.

[2] Goenka AH, Shah SN, Remer EM (2012). Imaging of the retroperitoneum. Radiol Clin North Am.

[3] Bernasconi JI, Mazzucco MR (2018). Target sign in neurogenic tumors. Rev Argent Radiol.

[4] Crist J, Hodge JR, Frick M, Leung FP, Hsu E, Gi MT, Venkatesh SK (2017). Magnetic resonance imaging appearance of schwannomas from head to toe: a pictorial review. J Clin Imaging Sci.

[5] Debaibi M, Essid R, Sghair A, Zouari R, Sahnoun M, Dhaoui A, Chouchen A (2022). Retroperitoneal schwannoma: uncommon location of a benign tumor. Clin Case Rep.

[6] Kuriakose S, Vikram S, Salih S, Balasubramanian S, Mangalasseri Pareekutty N, Nayanar S (2014). Unique surgical issues in the management of a giant retroperitoneal schwannoma and brief review of literature. Case Rep Med.

[7] Li S, Wu Z, Lin X, Ye L, Zeng X (2025). Retroperitoneal schwannoma: a clinicopathological analysis of 14 cases. Ann Diagn Pathol.

[8] Singh M, Kumar L, Chejara R, Prasad OP, Kolhe Y, Saxena A (2014). Diagnostic dilemma of a rare, giant retroperitoneal schwannoma: a case report and review of literature. Case Rep Oncol Med.

[9] Xiao J, Cai L, Pu J, Liu W, Jia C, He X (2022). Clinical characteristics and prognosis of cystic degeneration in retroperitoneal schwannoma: a retrospective study of 79 patients. Cancer Med.

[10] Radojkovic M, Mihailovic D, Stojanovic M, Radojković D (2018). Large retroperitoneal schwannoma: a rare cause of chronic back pain. J Int Med Res.

[11] Liu QY, Lin XF, Zhang WD, Li HG, Gao M (2013). Retroperitoneal schwannomas in the anterior pararenal space: dynamic enhanced multi-slice CT and MR findings. Abdom Imaging.

[12] Al-Dasuqi K, Irshaid L, Mathur M (2020). Radiologic-pathologic correlation of primary retroperitoneal neoplasms. Radiographics.

[13] Mota MM, Bezerra RO, Garcia MR (2018). Practical approach to primary retroperitoneal masses in adults. Radiol Bras.

[14] Gulati V, Swarup MS, Kumar J (2022). Solid primary retroperitoneal masses in adults: an imaging approach. Indian J Radiol Imaging.

[15] Millan Cortés C, Ayala Perez C (2022). Schwannoma retroperitoneal como diagnóstico diferencial de tumor retrocavo, reporte de un caso. Rev Cir.

[16] Ji JH, Park JS, Kang CM, Yoon DS, Lee WJ (2017). Laparoscopic resection of retroperitoneal benign neurilemmoma. Ann Surg Treat Res.

[17] Goh BK, Tan YM, Chung YF, Chow PK, Ooi LL, Wong WK (2006). Retroperitoneal schwannoma. Am J Surg.

[18] Ahmed KB, Mallat F, Hmida W, Chavey SO, Abdallah AB, Tlili K (2014). Huge retroperitoneal schwannoma in a young male. Int J Case Rep Images.

